# Quality of life in Wilson's disease

**DOI:** 10.4103/0972-2327.40224

**Published:** 2008

**Authors:** R. N. Komal Kumar, A. B. Taly, K. P. S. Nair, S. Sinha, L. K. Prashanth, N. Vidya, G. R. Arunodaya, S. Rao

**Affiliations:** Department of Neurology, National Institute of Mental Health and NeuroSciences (NIMHANS), Bangalore, India; 1 Department of Psychiatric and Neurological Rehabilitation, National Institute of Mental Health and NeuroSciences (NIMHANS), Bangalore, India; 2 Department of Biostatistics, National Institute of Mental Health and NeuroSciences (NIMHANS), Bangalore, India

**Keywords:** Quality of life, WHO-BREF, Wilson's disease

## Abstract

**Background:**

Assessment of Quality of life (QoL) is fast assuming significance as the measure of health in many disorders.

**Aim:**

To correlate clinical severity and QoL in patients with Wilson's disease (WD).

**Materials and Methods:**

We evaluated patients of WD on regular follow up for at least two years and aged over 18 years using Neurological Symptom Score (NSS) for clinical severity and WHO-BREF for QoL at a university teaching hospital. Patients with inability to respond to the questionnaire due to behavioral problems, low IQ or other disease related factors were excluded. These 30 patients (M:F:: 23:7) had a mean age of 27.97 ± 11.16 years at evaluation and the mean duration of treatment of 9.2 ± 6.4 years.

**Results:**

All four domains of WHO-QoL-BREF viz., Physical, Psychological, Social and Environmental correlated well with each other (*p* < 0.01). The NSS correlated inversely with the physical domain (*p* < 0.02), while the duration of treatment had a positive correlation with the physical domain (*p* < 0.01). None of the other features of QoL showed any significant correlation with age, NSS or duration of treatment.

**Conclusion:**

QoL is complementary to formal neurological assessment and should be routinely incorporated in the evaluation of outcome of patients with WD and other chronic neurological disorders.

## Introduction

In clinical research, there is a recent shift of trend from the assessment of impairment and disability to measurement of Quality of Life (QoL), since QoL reflects how well the individual is able to cope up in daily life with the burden of the disease and the treatment. QoL has been defined to generally correspond to the total well-being and encompasses both the physical and the psychological determinants such as emotional well being, behavioral competence, sleep and rest, energy and vitality and general life satisfaction.[1] Hence, the assessment of QoL becomes an important tool for neurological disorders that require long-term treatment, since the therapeutics, functional abilities and interaction of the individual with the environment and society must be addressed.

Wilson's disease (WD) is a rare metabolic disorder that leads to the accumulation of copper in tissues and multiple system involvement, the most prominent being liver and brain. A genetic defect at chromosome 13q14.3 results in defective transport of copper across membranes. The deposition of copper in liver occurs early and causes progressive hepatic dysfunction and portal hypertension. Excess copper in brain cells results in neuronal injury and produces motor, intellectual and behavioral dysfunctions. Untreated patients have progressive course and premature death but regular treatment with decoppering agents results in the significant remission of signs and symptoms. Logically, this should result in improvement in the QoL after treatment. However, these patients require taking medication for life long, making frequent visits to hospitals for follow-up and needing to undergo repeated laboratory tests to monitor disease, therapy and side effects of medications. These factors may affect their QoL. A literature search of Pub med with key words “Wilson's disease” and “Quality of Life” did not reveal many reports.[2] Collie in 2005 had addressed the impairment of quality of life in patients with liver disease and cognitive dysfunction and emphasized the need of early diagnosis.[3] This study, therefore, has addressed the issue of QoL in a cohort of patients with WD who were on regular treatment and follow-up at a tertiary care facility.

## Materials and Methods

### Subjects

Patients were selected from a large cohort of WD, followed at a tertiary care University teaching hospital, from south India. The diagnosis of WD was established by clinical features, presence of Kayser-Fleischer (KF) Ring on slit lamp examination, low serum ceruloplasmin and copper and increased 24 h urinary copper excretion. Inclusion criteria for the study were the definite diagnosis of WD and regular follow up for at least 2 years or more and age over 18 years. Patients who were unable to answer the questionnaire due to behavioral problems, low IQ or other disease related factors *per se* were excluded.

### Methods

#### World Health Organization QoL brief questionnaire (WHO-QoL-BREF)

QoL was assessed using self-reported World Health Organization QoL brief questionnaire (WHO-QoL-BREF)[4] [Appendix]. The WHO-QoL-BREF contains one question from each facet relating to QoL, viz., physical, psychological, social and environment relationships and two questions from overall QoL and general health facets of WHOQOL-100 totaling to 26 questions. Each individual assessment has a range of 1-5 where one is worst and five is best score. The questionnaire contains three negatively phrased items and the scoring for these questions were recomputed at the time of analysis.

#### Neurological symptom score

Neurological impairment and disability were assessed using Neurological Symptom Score (NSS). This score assesses the neurological status of the patient using numerical grade to various neurological signs[5] and includes 14 neurological parameters: speech (0-5), eye movements (0-3), sialorrhea (0-3), deglutition (0-4), bradykinesia (0-3), rigidity (0-3), dystonia (0-3), tremor (0-4), chorea (0-4), dysdiadokinesis (0-3), plantar response (0-1), muscle stretch reflexes (0-2), postural instability (0-4) and gait (0-4). The score ranges from 0 to 46, where zero is the best score and 46 indicates severe disability.

### Statistics

Statistical analysis was performed using SPSS v10. Mean, range and standard deviation were noted for the continuous variables. The statistical correlation between different domains of QoL and clinical parameters was performed using Pearson Correlation Coefficient test. Pearson correlation coefficient is the measure of linear association between two variables and the values range form −1 to + 1. The sign of coefficient indicates the direction of the relationship and its absolute value indicates the strength, with larger absolute values indicating stronger relationships. The values were considered significant if *p* ≤ 0.05.

## Results

Thirty patients (M:F:: 23:7) with WD who fulfilled the inclusion criteria were recruited for this study. These patients were on regular decoppering therapy, viz., zinc sulfate and penicillamine for a variable period. Their mean age at the time of evaluation was 27.97 ± 11.16 years (range: 18-62 years). The mean age at onset and diagnosis was 16.8 ± 7.1 years and 20.3 ± 10.7 years, respectively. Hence, the delay in diagnosis was 4.07 ± 6.1 (range: 1 month to 22 years). The mean duration of illness was 11.08 ± 9.31 years, (range: 2-49 years) and duration of treatment was 9.2 ± 6.4 years (range: 2-31 years). All patients except one were from lower socioeconomic status with an annual income of less than INR 20,000 (440 US dollars). Twenty-three patients had neurological involvement, two each had psychiatric, musculoskeletal and multiple system involvement and one patient had predominant hepatic involvement.

The median NSS score was 3 (range: 0-12). The mean scores of different WHO-QoL-BREF domains were as follows: physical (3.65 ± 0.55), psychological (3.53 ± 0.75), social relations (3.93 ± 0.95) and environmental (3.47 ± 0.62). All four domains of QoL effectively correlated with each other [Table 1]. Table 2 shows the relationship between WHO-QoL-BREF domain scores and age, the duration of treatment and NSS reflecting severity of disease. The NSS correlated inversely with the physical domain of QoL. The physical domain had a significant correlation with the duration of treatment (*p* < 0.01) and NSS (*p* < 0.05). None of the other domains showed any significant correlation with the duration of treatment and NSS. Age did not correlate with any of the domains of QoL [Table 2].

**Table 1:** Pearson correlation coefficient for quality of life domains

	**Physical**	**Psychological**	**Social relationship**	**Environmental**
Physical				
Correlation coefficient	1.000	0.475**	0.640**	0.558**
P	-	0.008	0.0001	0.001
Psychological				
Correlation coefficient	0.475**	1.000	0.441*	0.537**
P	0.008	-	0.015	0.002
Social relationship				
Correlation coefficient	0.640**	0.441*	1.000	0.638**
P	0.0001	0.015	-	0.0001
Environmental				
Correlation coefficient	0.558**	0.537**	0.638**	1.000
P	0.001	0.002	0.0001	-

** Correlation is significant at the 0.01 level (2-tailed);

* Correlation is significant at the 0.05 level (2-tailed)

**Table 2:** Pearson correlation coefficient for clinical parameters and quality of life domains

**Parameter**	**Physical**	**Psychological**	**Social relationship**	**Environmental**
Age at evaluation				
Correlation coefficient	0.091	0.227	-0.056	-0.092
P	0.633	0.228	0.769	0.629
Duration of treatment				
Correlation coefficient	0.469**	0.283	0.335	0.220
P	0.009	0.13	0.07	0.244
NSS†				
Correlation coefficient	-0.453*	-0.229	-0.346	-0.355
P	0.012	0.223	0.061	0.054

** Correlation is significant at the 0.01 level (2-tailed);

* Correlation is significant at the 0.05 level (2-tailed);

† Neurological symptom score

## Discussion

The traditional assessment in chronic neurological disorders focuses on impairment, disability and survival. This approach ignores the perceptions of the patients. The efficacy and acceptability of any intervention depends upon benefits perceived by the recipient. A wide variety of research designs have been used in QoL research through the years. The number of different instruments or combinations of instruments nearly equals the number of studies conducted. This is primary due to conceptual difficulties and the terminology used by researchers.[6] The Development of WHO quality of life (WHO QoL) scale was required for a genuine internationally accepted QoL assessment. It is an abbreviated version of the WHOQoL-100, developed by WHOQoL group. The WHOQoL-100 encompasses 26 facets that are universally regarded as important in assessing the QoL and four general questions that address over all QoL and health. Four questions regarding each facet are included. The 26 facets were appropriately grouped into four domains: physical, psychological, social relationships and environment. The problem of cultural comparability has been explicitly dealt with in the development of the 100-item WHO QoL. The tool was developed simultaneously across a broad range of member countries, assuring that it could be used more multiculturally and multilingually than any other existing quality of life tool. While the WHOQOL-100 allows a detailed assessment of individual facets relating to quality of life, it is too lengthy to administer for studies where QoL is only one variable of interest. In these instances, assessments will be more willingly incorporated if they are brief, convenient and accurate. Hence, an abbreviated version of the WHOQoL-100, the WHO-QoL-BREF was developed. The WHO-QoL- BREF has been shown to assess adequately domains relevant to QoL in a large number of cultures worldwide. Domain scores produced by the WHO-QoL-BREF have been shown to correlate at around 0.9 with the WHOQoL-100 domain scores, which has itself demonstrated the criterion validity. They have also been shown to display good discrimination validity (ability in discrimination between ill and well respondents), content validity and test - retest reliability. Having an international quality of life assessment such as the WHOQoL makes it possible to carryout QoL research collaboratively in different cultural settings and to directly compare the results obtained in these different settings, hence used in our study.

The concept of QoL is multidimensional and reflects how well an individual can cope with the burden of the disease and treatment. It was defined as the individual's perception of their position in life in the context of the culture and value systems in which they live and in relation to their goals, expectations, standards and concerns. It is a broad ranging concept, that incorporates the following in a complex manner: individual's physical health, psychological state, level of independence, social relationships, personal beliefs and their relationships to salient features of the environment.[7] This definition highlights the view that QoL is subjective, includes both positive and negative facets of life and is multidimensional. The QoL of an individual depends on functional abilities and the interaction with the environment and society. Wilson's disease produces a wide range of neurological symptoms and signs and can serve as a useful model for chronic disabling disorders. Wilson's disease is an imminently treatable condition and if recognized early it has a very good clinical outcome and impact on QoL. However, occasionally, despite early diagnosis and treatment some patients have a relentlessly progressive course and premature death. Prashanth *et al.* concluded that around half the patients with severe form of WD improved clinically and radiologically with decoppering therapy.[8]

Currently, QoL is being used as a measure to determine both the impact of various neurological disorders on life of individuals and the effectiveness of treatment and rehabilitation measures. In a study of patients with multiple sclerosis, higher Expanded Disability Status Score (EDSS) levels were associated with impaired QoL.[9] Paul *et al*.[10] observed that QoL was negatively affected by myasthenia gravis with notable differences across domains of functioning sensitive to physical limitation, including ability to engage in physical activities.

In the present study, all domains of QoL co-related with each other [Table 1] indicating that QoL domains are interlinked and provide a more holistic approach to health. Individuals with limited functional mobility and the ability to interact within their environment and society will most likely perceive their QoL to be poor.

Physical QoL was negatively affected by NSS (*p* < 0.01). The higher NSS score means more severe physical problems that would limit the mobility and activity of daily living and is perceived by subjects as poor physical QoL. Physical domain positively correlated with duration of treatment [Table 2]. The symptoms of WD respond very well to treatment and subjects on treatment for longer duration may have lesser physical symptoms and signs. The physical domain is the only domain affected by duration and severity of disease. The psychological, social and environmental domains of QoL did not relate to severity of disease or duration of symptoms. Further research is required to understand what determines various domains of QoL in subjects with WD. This may help in planning and delivery of services to patients.

Research with regard to QoL in persons with WD is virtually non-existent. Sutcliffe *et al*.[2] followed 24 patients of WD who underwent liver transplantation between 1988 to 2000. They used 36-Item Short Form 36 Health Survey Questionnaire to assess the QoL among survivors and compared it with the controls and observed that liver transplantation can be safely performed in patients with WD, with excellent long-term results and QoL. It is a self-report generic measure questionnaire containing 36 items organized into 8 domains that cover a wide range of physical activities and psychosocial perceptions including global ratings of general health. However, using WHO QoL, an international quality of life assessment, which has exclusively handled the problem of cross-cultural comparability can make it possible to carryout and compare the QoL research across the world. There are several aspects that have to be studied with regard to QoL from both the cross-sectional and longitudinal studies. The successful management of WD requires more than provision for medical treatment.

Most of the patients with WD were in their third decade and were on regular treatment. According to inclusion criteria, only the selected patients were able to answer the questions independently or were above 18 years of age, thus introducing a bias towards a more stable group of patients. The subjects who were able to understand and able to respond to questionnaire were included and patients who were unable to do so due to disease process per se were not evaluated and hence the QoL in these severely affected patients is likely to be worse and not reflected in the study.

The present study has a few limitations because of the convenient sampling. The data obtained from the patients were not compared with those from normal healthy controls. The sample size is small but WD being a rare disease; it is difficult to have larger numbers in a short study period. Nevertheless, our study adds a new dimension to the assessment of outcome of WD. It strengthens the importance of regular and life-long medications in this group. There is a need to incorporate measures such as QoL in addition to traditional scales of impairment to assess the impact of WD and its treatment on life of these patients.
